# The Fruits of Vivisection

**Published:** 1892-10-29

**Authors:** Andrew Clark

**Affiliations:** President of the Royal College of Physicians


### THE FRUITS OF VIVISECTION.

By Sir Andrew Clark, Bart., M.D., F.R.S., President of the Royal College of Physicians.

[At our request, Sir Andrew Clark has furnished us with the following notes on the burning questions of the utility and morality of Vivisection. The best use, we think, which we can make of this important contribution to a painful controversy is to print it in full, and to give it as wide a circulation as possible. It is proper to say that the notes, though necessarily hurriedly prepared, express the mature convictions of their author.]

The law of vicarious sacrifice is a law of the physical, mental, moral, and spiritual life; and no living sentient being can escape from its operation. It is plainly the appointment of God, and if one animal may not be used in the service of another and higher animal God is neither just nor loving.

Experiments performed upon animals with due care, for a high purpose, in a reverent spirit, and with results in good transcending any suffering inflicted, are not cruel. Cruelty lies in the wanton infliction of suffering and in the performance of experiments for a low end, or carelessly designed, or barren in results of good justifying the suffering, if any, which has been inflicted.

It is folly now to argue the question whether experimental research has contributed to the advancement of theoretical and practical knowledge, for it is certain that no substantial advance has been made in physiological knowledge; and none in the departments of midwifery, surgery, and medicine without the assistance of this method of research.

Have we the right to cause other creatures to suffer in our service? The right has been always and everywhere assumed and practised.

We inflict suffering upon animals for our convenience and use; we do so for our pleasures, we do so for the gratification of our vanity. And in the pursuit of the balance of power in Europe we have sacrificed the lives of hecatombs of men and ruined the happiness of countless families.

Why, then, may we not experiment upon animals for the redemption of animals, for the relief of human suffering, and for the advancement of knowledge which is the natural revelation of God?

The discovery of the circulation of the blood by Harvey through "many dissections of living things," and by "frequently looking into many and various living animals," conferred the same service upon practical medicine that the law of gravitation conferred upon practical physics.

At one time in Vienna the deaths from childbed fever amounted to 16 per cent. By long and patient study, assisted by experiment upon animals, the mortality was reduced to under 1 per cent.

Compare an amputation in the time of Ambrose Paré with an amputation and its results in the present day. Consider the hæmorrhages, the sloughings, the long ulcerations, the stump neuralgias, the constitutional fevers, and the high mortality of the cases in those days. But now all these things have been banished almost entirely through the knowledge acquired by experiments upon living animals. There was a time, still within living memory, when the operations of ovariotomy was declared by some of the leading surgeons to be unjustifiable. But now, by the help of experimental research, ovariotomy has become one of the most successful operations in surgery; and it has been the privilege of one man by its aid to restore to health six hundred women, who, if left alone, would have died.

By this same experimental research surgeons also have been enabled to arrest or cure diseases by excising portions of the stomach, by removing one of the kidneys, by amputating the larynx, and by extirpating portions of the brain and spinal cord.

By experimental research Koch discovered in the presence and action of the tubercular bacillus, the true course of tuberculous or consumptive disease; and this discovery has not only thrown a flood of light upon the natural history of consumption, but has enabled us to discover its presence when otherwise unsuspected, and to bring about a successful revolution in the treatment of surgical tuberculosis. Furthermore, there are just reasons for expecting that the continuance of this experimental research will lead to the discovery of means for the prevention, and, perhaps, even the cure of pulmonary consumption, which destroys so many of the fairest and best of our race.

By experimental research we have discovered the condition for using with efficiency and safety almost all the stronger and most useful drugs, such as digitalis chloroform, ether, chloral, nitrate of amyl, nitroglycerine, and many others.

By experiment upon animals we have discovered the nature and relations of infectious diseases; and we have learned how in some measure to prevent the development and to control the spread of fevers, cholera, anthrax, and septicaemia. Through experiments on animals we have received the use of the electric telegraph and all the various services which electricity now renders to the convenience and uses of man. And yet with all these services before us, one cannot scratch the neck of a rabbit for the advancement of knowledge without becoming a legal criminal. But on the other hand, for your pleasure, or for your profit, or for any other object than the promotion of knowledge, you may, without let or hindrance, beat, starve, mutilate, or destroy as many animals as you please.

Knowledge can be advanced now only by experiment, and if experiment is to be suppressed, and if consistently the use of knowledge acquired in this manner is to be rejected, then it is certain that the art of preventing and of curing the diseases of beasts and men will decay; that physical and mental degeneration will follow, and that science in England will become the contempt and pity of the world.

And if physiology, pathology, and therapeutics were to continue their course without the help of experiments upon animals, the errors that would be committed in the exercise of our art would bring about greater suffering and greater unhappiness than all the vivisections which have ever been performed.

Lastly, if experimental research hardens the hearts of experimenters, it is only too plain that an active antagonism to it begets a disregard of accuracy, a violation of charity, and a spirit of calumny which have no parallel among ordinary men.

